# Clarithromycin enhances bortezomib-induced cytotoxicity via endoplasmic reticulum stress-mediated CHOP (GADD153) induction and autophagy in breast cancer cells

**DOI:** 10.3892/ijo.2011.1317

**Published:** 2011-12-23

**Authors:** SEIICHIRO KOMATSU, KEISUKE MIYAZAWA, SHOTA MORIYA, AKIKO TAKASE, MUNEKAZU NAITO, MASATO INAZU, NORIO KOHNO, MASAHIRO ITOH, AKIO TOMODA

**Affiliations:** 1 Department of Breast Oncology, Tokyo Medical University, Tokyo 160-8402, Japan; 2 Department of Biochemistry, Tokyo Medical University, Tokyo 160-8402, Japan; 3 Department of Anatomy, Tokyo Medical University, Tokyo 160-8402, Japan; 4 Institute of Medical Science, Tokyo Medical University, Tokyo 160-8402, Japan

**Keywords:** bortezomib, clarithromycin, breast cancer, ER-stress, autophagy, CHOP

## Abstract

The specific 26S proteasome inhibitor, bortezomib (BZ) potently induces apoptosis as well as autophagy in metastatic breast cancer cell lines such as MDA-MB-231 and MDA-MB-468. The combined treatment of clarithromycin (CAM) and BZ significantly enhances cytotoxicity in these cell lines. Although treatment with up to 100 μg/ml CAM alone had little effect on cell growth inhibition, the accumulation of autophagosomes and p62 was observed after treatment with 25 μg/ml CAM. This result indicated that CAM blocked autophagy flux. However, the combined treatment of BZ and CAM resulted in more pronounced autophagy induction, as assessed by increased expression ratios of LC3B-II to LC3B-I and clearance of intracellular p62, than treatment with BZ alone. This combination further enhanced induction of the pro-apoptotic transcription factor CHOP (CADD153) and the chaperone protein GRP78. Knockdown of CHOP by siRNA attenuated the death-promoting effect of BZ in MDA-MB-231 cells. A wild-type murine embryonic fibroblast (MEF) cell line also exhibited enhanced BZ-induced cytotoxicity with the addition of CAM, whereas a *Chop* knockout MEF cell line completely abolished this enhancement and exhibited resistance to BZ treatment. These data suggest that endoplasmic reticulum (ER)-stress mediated CHOP induction is involved in pronounced cytotoxicity by combining these reagents. Simultaneously targeting two major intracellular protein degradation pathways such as the ubiquitin-proteasome system by BZ and the autophagy-lysosome pathway by CAM may improve the therapeutic outcome in breast cancer patients via ER-stress mediated apoptosis.

## Introduction

Approximately 75% of breast cancer is categorized as estrogen receptor α-positive, with estradiol-bound estrogen receptor α as the key determinant in promoting breast cancer growth (1). Blocking the action of estrogen receptor α by selective estrogen receptor modulators, such as tamoxifen, has been the most common treatment strategy for breast cancer patients (2). However, despite the significant benefits of tamoxifen treatment, almost all patients with metastatic disease and as many as 40% of the patients receiving adjuvant tamoxifen therapy do not respond, or acquire resistance during treatment (3). Additionally, in estrogen receptor α-negative breast cancer patients, therapeutic outcome for anthracycline-based chemotherapy is unsatisfactory (4). Therefore, novel strategies are required to improve therapeutic efficacy for these breast cancer patients.

Bortezomib (BZ), a selective and potent inhibitor of the 26S proteasome, has been approved for treating multiple myeloma (5). Increasing evidence indicates that inhibition of the 26S proteasome by BZ leads to the accumulation of misfolded proteins in the endoplasmic reticulum (ER), resulting in ER stress followed by a coordinated cellular response known as unfolded protein response (UPR) (6–10). Downstream effectors of UPR include activation of the chaperone protein GRP78 (Bip) to maintain ER integrity, and the transcription factor CHOP (the C/EBP homologous protein, also designated as GADD153) to mediate cell death when ER stress is beyond the tolerance of the cell adaptation (11–13). Many *in vitro* studies have demonstrated that BZ potently induces cell growth inhibition and apoptosis in breast cancer cell lines via UPR (14–16). However, clinical trials using BZ as a single agent for treating metastatic breast cancer were initially disappointing (17). A new study combining the pure anti-estrogen fulvestrant with BZ suggested that the combination of anti-estrogen therapies with proteasome inhibition might increase treatment efficacy in estrogen receptor α-positive-breast cancer cell lines (18). It was also reported that BZ inhibited breast cancer cell growth and reduced osteolysis by down-regulating metastatic genes in xenograft mice (19).

Macroautophagy (hereafter, autophagy) is a highly conserved cellular process in eukaryotes. Intracellular proteins and organelles including ER are engulfed in a double-membrane vesicle called an autophagosome and delivered to lysosomes for degradation by lysosomal hydrolases (20,21). Autophagy occurs in parallel with the ubiquitin-proteasome system that is specific to short-lived protein turnover (22). Autophagy has been regarded as a bulk non-selective degradation system for long-lived proteins and organelles. However, recent reports focused on the selective degradation pathway of ubiquitinated protein through autophagy via p62 and the related protein NBR1, which are docking proteins having both a microtubule-associated protein 1 light chain 3 (LC3)-interacting region and a ubiquitin-associated domain (23). LC3 is essential for autophagy and is associated with autophagosome membranes after processing (24). Thus, by binding ubiquitin via their C-terminal ubiquitin-associated domains, p62-mediated degradation of ubiquitinated cargo occurs by selective autophagy. The two major intra-cellular degradation systems are thus directly linked (22,23).

We previously reported that combined treatment with BZ and bafilomycin A_1_, resulted in synergistic enhancement of the cytocidal effect along with the induction of ER stress in myeloma cells (9). Bafilomycin A_1_ is a macrolide antibiotic, a specific inhibitor of vacuolar-ATPase, and is used as an autophagy inhibitor in the late stage of this process (25). A recent report demonstrated that clarithromycin (CAM), a semi-synthetic macrolide anti-biotic derived from erythromycin, inhibited autophagy flux and exhibited some cell growth inhibition in myeloma cells (26). High efficacy of the chemotherapeutic regimen combining CAM with lenalidomide, a derivative of thalidomide, in treating myeloma was recently reported (27,28). Many lines of evidence indicate that certain macrolide antibiotics exert some anti-tumor activities in marginal zone B-cell lymphoma, leukemia, non-small lung cancer, and melanoma (29–34). Although the underlying molecular mechanism has not yet been clarified, this anti-tumor activity does not appear to be mediated by eradication of *Helicobacter pylori*, as with MALT lymphoma (35). A direct apoptosis inducing-effect of CAM has been observed in lymphoma cells (36).

Based on previous data, we attempted to investigate whether combined treatment with CAM and BZ for simultaneously inhibit of two major intracellular protein degradation systems enhances ER stress-mediated cell death in breast cancer cells.

## Materials and methods

### Reagents

BZ was purchased from Toronto Research Chemical Inc. (North York, Ontario, Canada). BZ was dissolved in dimethyl sulfoxide (DMSO) at a concentration of 1 mM as a stock solution. CAM, purchased from Wako Pure Chemical Industries, Ltd. (Osaka, Japan), was dissolved in ethanol at a concentration of 5 mg/ml as a stock solution. E-64d and Pepstatin A, which are inhibitors of lysosomal proteases, were purchased from Biomol International LP (Plymouth Meeting, PA, USA).

### Cell lines and culture conditions

For this study, we used the breast cancer cell lines MDA-MB-231 and MCF7 (kind gifts of Dr Keiichi Iwaya, Department of Basic Pathology, National Defense Medical College, Saitama, Japan), and the MDA-MB-468 cells, obtained from the American Type Culture Collection (ATCC) (Manassas, VA). A CHOP^−/−^ MEF cell line (CHOP-KO-DR) established from a 13.5-day-old CHOP^−/−^ mouse embryo by SV-40 immortalization and a CHOP^+/+^ MEF cell line (DR-wild-type) established by SV-40 immortalization as a control cell line for CHOP-KO-DR were obtained from ATCC (Rockville, MD). MDA-MB-231, MDA-MB-468, and MCF7 cells were maintained in continuous culture in RPMI-1640 medium (Gibco, Grand Island, NY) supplemented with 10% FBS (PAA Laboratories, Austria), 2 mM L-glutamine, penicillin (100 U/ml) and streptomycin (100 μg/ml) (Wako Pure Chemical Industries, Ltd.). CHOP-KO-DR and DR-wild-type cells were maintained in Dulbecco’s modified Eagle’s medium (Sigma, St. Louis, MO) supplemented with 10% FBS, penicillin (100 U/ml), and streptomycin (100 μg/ml). All cell lines were cultured in a humidified incubator containing 5% CO_2_ and 95% air at 37°C.

### Assessment of the viable number of cells among cultured cells

The number of viable cells was assessed by CellTiter Blue, a cell viability assay kit (Promega Corp., Madison, WI), with fluorescence measurements at 570 nm for excitation and 590 nm for fluorescence emission.

### Morphology assessment

After trypsinization, cell suspensions were sedimented and fixed on slide glasses using a Shandon Cytospin II (Shandon, Pittsburgh, PA). Preparations were then stained with May-Grünwald-Giemsa, and examined using a digital microscope BZ-9000 (Keyence Co., Osaka, Japan).

### Immunoblotting

Immunoblotting was performed as previously described (9). Cells were lysed with RIPA lysis buffer (Nacalai Tesque Inc., Kyoto, Japan) containing 1 mM PMSF, 0.15 U/ml aprotinin, 10 mM EDTA, 10 mg/ml sodium fluoride, and 2 mM sodium orthovanadate. Cellular proteins were quantified using a DC Protein Assay Kit of Bio-Rad (Richmond, CA). Equal amounts of proteins were loaded onto the gels, separated by SDS-PAGE, and transferred onto Immobilon-P membrane (Millipore Corp., Bedford, MA). The membranes were probed with first antibodies (Abs) such as anti-LC3B Ab (Novus Bioloicals, Inc., Littleton, CO), anti-p62 mAb (sequestsome-1) (Santa Cruz, CA), anti-phospho-JNK Ab (Thr183/Tyr185) (Cell Signaling Technology, Danvers, MA), anti-cleaved caspase-3 Ab (Asp175) (Cell Signaling Technology), anti-phospho-eIF2α Ab (Ser51) (Cell Signaling Technology), and anti-GAPDH mAb (Santa Cruz). Immunoreactive proteins were detected with horseradish peroxidase-conjugated second Abs and an enhanced chemiluminescence reagent (ECL) (Millipore). Densitometry was performed using a Molecular Imager, ChemiDoc XRS System (Bio-Rad).

### Gene expression analysis

Total RNA was isolated from cell pellets using Isogen (Nippon Gene, Tokyo, Japan) and genomic DNA was removed using RQ1 RNase-Free DNase (Promega) at 37°C for 30 min, followed by extraction with phenol chloroform and ethanol precipitation. Reverse-transcription using a PrimeScript RT Master Mix (Takara Bio Inc. Ohtsu, Japan) was performed according to the manufacturer’s instructions. Real-time PCR was performed on 3 ng of cDNA using validated SYBR Green gene expression assays for human *GRP78, CHOP, GADD34* and *p62* in combination with SYBR Premix Ex Taq II (Takara Bio Inc.). The primer for *p62* was: forward 5′-AGCTGCCTTGTACCCACATC-3′. Reverse 5′-CAGAG AAGCCCATGGACAG-3′. The sequences of primes for *GRP78, CHOP, GADD34* and *GAPDH* were as we previously described (9).

Quantitative real-time PCR was performed in duplicates in a Thermal Cycler Dice Real Time System TP800 (Takara Bio Inc.) with the following conditions: initial cDNA denaturation at 95°C for 30 sec, followed by 45 cycles of the sequence of denaturation at 95°C for 5 sec and simultaneous annealing and extension at 60°C for 30 sec. The data were analyzed using Thermal Cycler Dice Real Time System Software (Takara Bio Inc.), and the comparative *C_t_* method (2^−ΔΔ^*^C^_t_*) was used for relative quantification of gene expression. The data of real-time PCR products were standardized to *GAPDH* as an internal control. To confirm the specific amplification of target genes, each gene product after real-time PCR was further separated by 1.5% agarose gel to detect a single band at the theoretical product size, as well as analysis of the dissociation curve for detecting a single peak.

### Electron microscopy

Electron microscopy was performed as previously described (37). *Transfection of CHOP siRNA.* For the gene silence of *CHOP* in MDA-MB231 cells, CHOP siRNA and control scramble siRNA, whose sequences are described below, were diluted to a final concentration of 20 nM in Opti-MEM I (Invitrogen, Paisley, UK), and transfection was performed with cells at 50% confluency using Oligofectamine transfection reagent (Invitrogen) according to the manufacturer’s instructions. CHOP sense: CUGAUUGACCGAAUGGUGATT. CHOP antisense: UCACCAUUCGGUCAAUCAGTT. Control sense: GACUACUGGUCGUUGAACUTT. Control antisense: AGU UCAACGACCAGUAGUCTT.

### Statistical analyses

The data are given as the mean ± SD. Statistical analysis was performed by using Mann-Whitney’s U test (two-tailed).

## Results

### Cell growth inhibition and apoptosis induction after treatment with BZ in breast cancer cell lines

**Figure 1:**
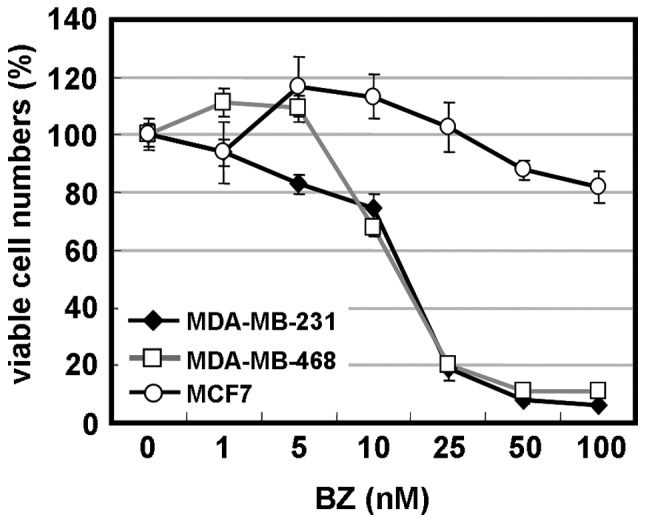

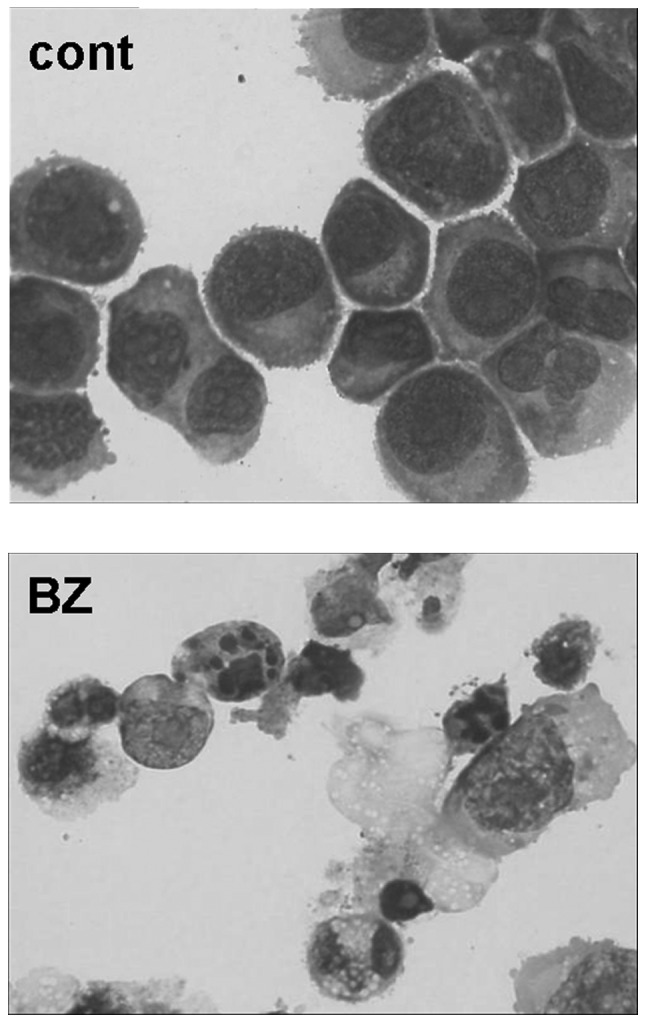

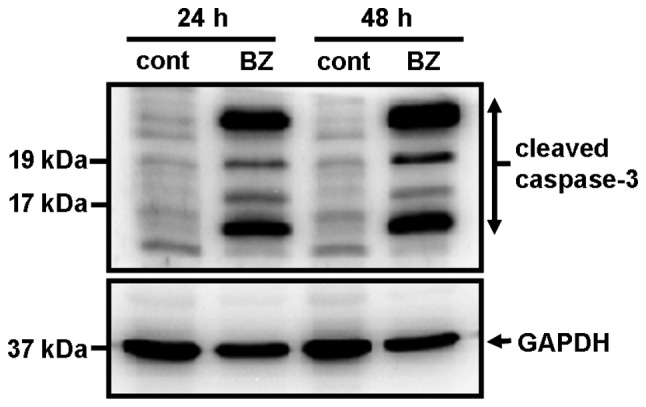
Cell growth inhibition after treatment with BZ in breast cancer cell lines. (A) MDA-MB-231, MDA-MB-468, and MCF7 cells were treated with BZ at various concentrations for 48 h. The viable cell number was assessed by CellTiter Blue as described in Materials and methods. (B) Morphological features after treatment with/without BZ (25 nM) for 48 h in MDA-MB-231 cells. May-Grünwald-Giemsa staining (original magnification ×1,000). (C) Immunoblotting with anti-cleaved caspase-3 Ab: MDA-MB-231 cells were treated with BZ (25 nM) for 24 and 48 h. Cells were lysed, and cellular proteins were separated by 15% SDS-PAGE and immunoblotted with anti-cleaved caspase-3 Ab. Immunoblotting with anti-GAPDH mAb was performed as an internal control.

MDA-MB-231 and MDA-MB-468 cells for estrogen receptor α-negative-breast cancer cell lines and MCF7 cells for an estrogen receptor α-positive breast cancer cell line were cultured in the presence of BZ at various concentrations. Viable numbers of cells were assessed after 48-h treatment. As indicated in Fig. 1A, BZ at concentrations of >10 nM exhibited potent cell growth inhibition of MDA-MB-231 and MDA-MB-468 cells, but was less effective in MCF7 cells. IC_50_ was 17 nM for MDA-MB-231 cells and 16 nM for MDA-MB-468 cells, which were almost equivalent to myeloma cell lines (9). Extended BZ-exposure time to 96 h indicated 50% cell growth inhibition at 25 nM in MCF7 cells (data not shown). Morphological studies confirmed chromatin condensation and nuclear fragments in MDA-MB-231 and MDA-MB-468 cells in response to BZ. However, some cells exhibited increased vacuoles in cytoplasm. Immunoblotting revealed the cleavage of caspase-3 after treatment with BZ (Fig. 1B and C). These data indicate that BZ induces apoptosis in MDA-MB-231 and MDA-MB-468 cells, as previously reported for various cell lines including myeloma (9).

### BZ induces autophagy in breast cancer cell lines

**Figure 2:**
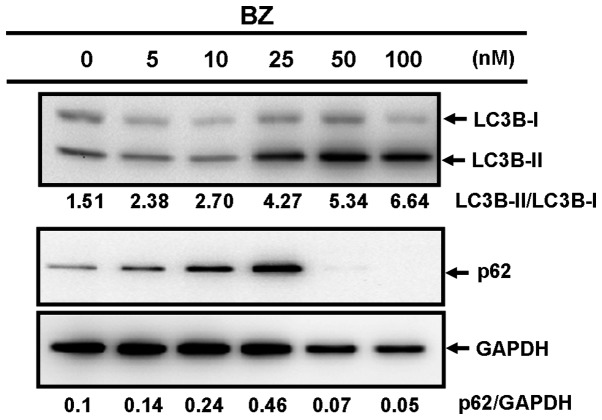

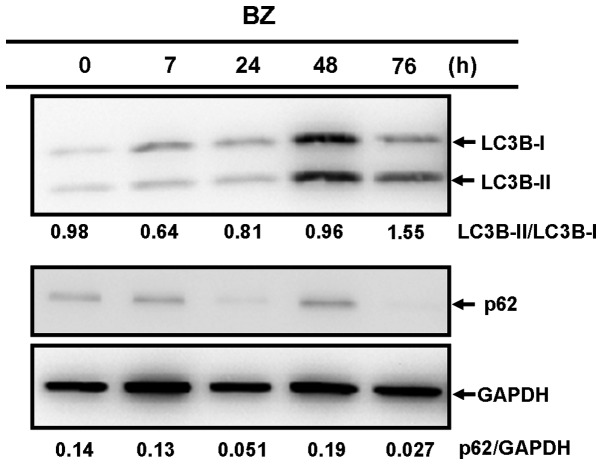

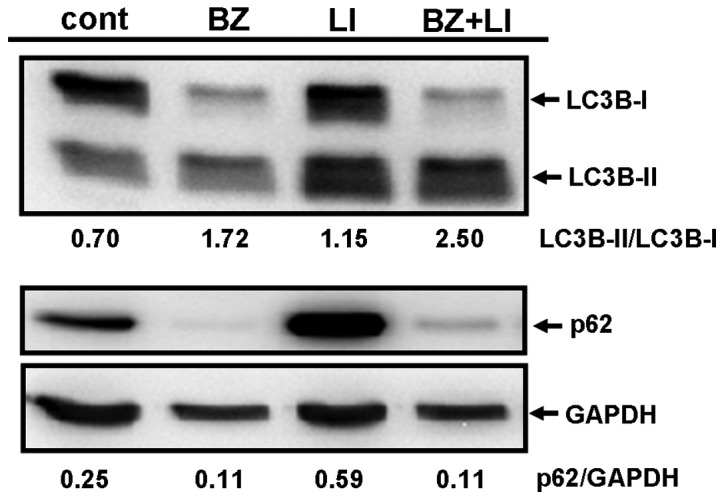
Autophagy induction in MDA-MB-231 cells after treatment with BZ. (A) MDA-MB-231 cells were treated with BZ at various concentrations for 48 h. Cellular proteins were separated by 15% SDS-PAGE for LC3B and 11.25% SDS-PAGE for p62. Immunoblotting was performed using anti-LC3B Ab and anti-p62 mAb. (B) MDA-MB-231 cells were treated with 25 nM of BZ for various lengths of time. Cellular proteins were separated by SDS-PAGE as described above and immunoblotted with anti-LC3B Ab and anti-p62 mAb. Immunoblotting with anti-GAPDH mAb was performed as an internal control. (C) MDA-MB-231 cells were cultured with/without BZ (25 nM) in the presence or absence of lysosomal inhibitors (LI), E-64d (10 μg/ml) and pepstatin A (10 μg/ml) for 48 h. Each number indicates the ratio of LC3B-II/LC3B-I and p62/GAPDH as determined by densitometry.

We previously reported that BZ induces autophagy, along with ER stress, in myeloma cells (9). LC3B exists in two cellular forms, LC3B-I and LC3B-II. LC3B-I is converted to LC3B-II by conjugation to phosphatidyl ethanolamine during the formation of autophagosomes. Therefore, the amount of LC3B-II is a good early marker of the formation of autophagosomes (24). Treatment with BZ induced increased ratios of LC3B-II/LC3B-I in a dose-dependent manner, whereas a significant reduction of p62 was observed after 76 h with BZ (Fig. 2A and B). Additionally, combined treatment with BZ and lysosomal inhibitors such as E-64d and pepstatin A, which were used to block the catabolic flux of the autophagic process, resulted in further enhancement of the LC3B-II/LC3B-I ratio, compared with treatment with BZ or lysosomal inhibitors alone (Fig. 2C). These data indicate that BZ induces autophagy, in agreement with previous reports on myeloma cells (9,24). However, it is noteworthy that an increased expression of p62 with 25 nM BZ for a 48-h treatment was observed (Fig. 2A and B).

### Combination of clarithromycin and BZ enhances cytotoxicity and autophagy

**Figure 3:**
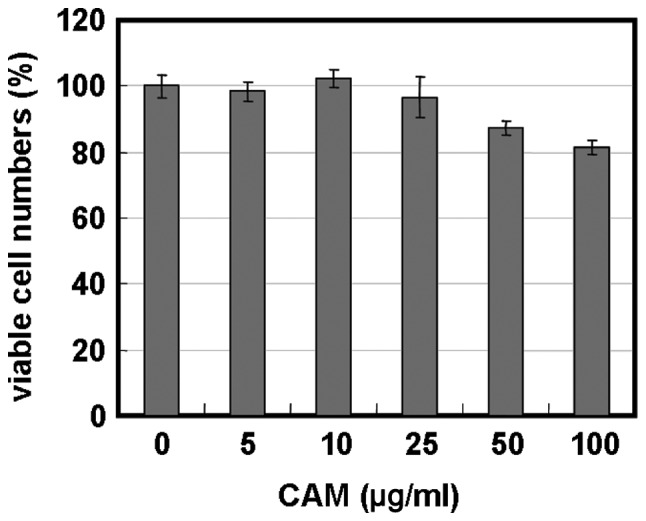

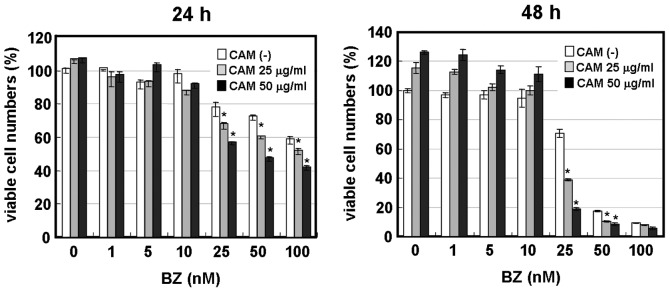

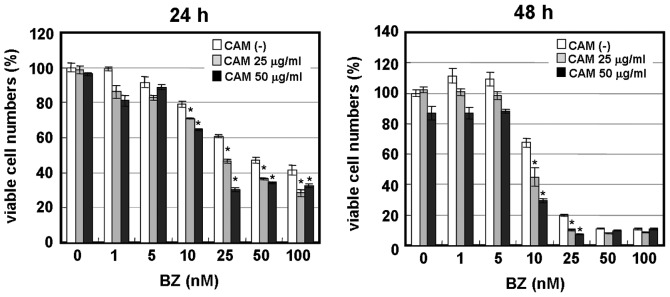
Cytotoxic effect on MDA-MB-231 and MDA-MB-468 cells after combined treatment with BZ plus CAM. MDA-MB-231 cells were cultured with CAM at various concentrations for 48 h (A). MDA-MB-231 cells (B) and MDA-MB-468 cells (C) were cultured with BZ at various concentrations for 24 and 48 h in the presence or absence of CAM at 25 and 50 μg/ml. The viable cell number was assessed as described in Materials and methods [^*^p<0.05, CAM (−) vs. CAM (+)].

**Figure 4:**
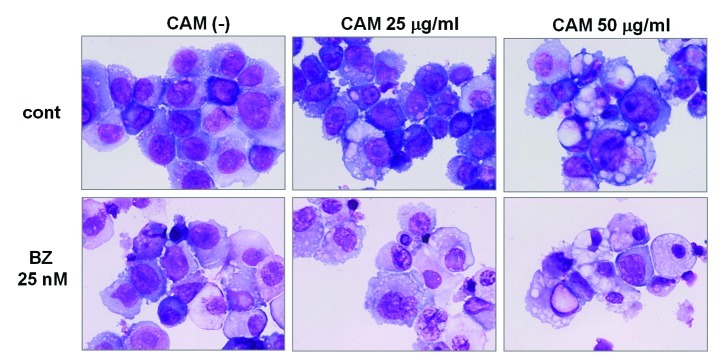

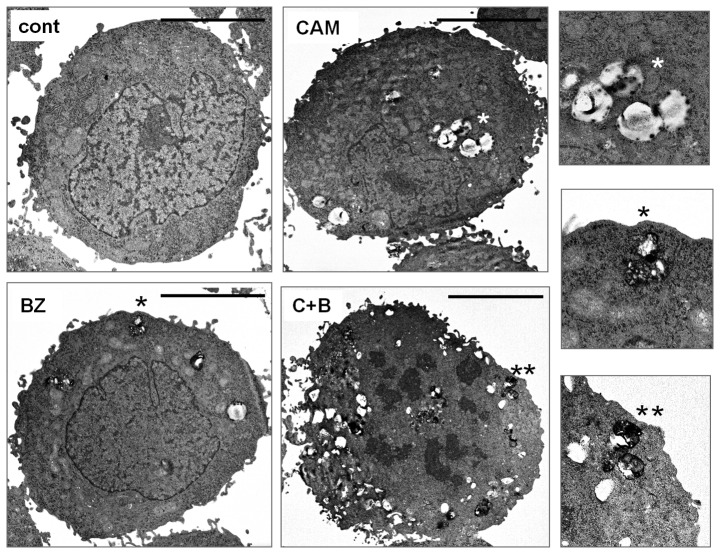
Morphological changes in MDA-MB-231 cells after treatment with BZ in the presence/absence of CAM. (A) May-Grünwald-Giemsa staining (original magnification ×1,000). MDA-MB-231 cell were cultured with/without BZ (25 nM) ± CAM (25 and 50 μg/ml) for 48 h. (B) Electron microscopy. MDA-MB-231 cells treated with/without BZ (25 nM) ± CAM (50 μg/ml) for 30 h. C + B indicates CAM + BZ. The scale bar in each panel indicates 10 μm.

It has been reported that CAM attenuates autophagy in myeloma cells (26). Although the mechanism still must be clarified, it is suggested that CAM may inhibit the latter part of autophagic flux, such as autolysosome formation and lysosomal hydrolysis (26). This results in an accumulation of autophagosomes in cytoplasm. Our recent study also demonstrated that combined treatment with BZ and bafilomycin A_1_ (BAF), which is a macrolide antibiotic that is used as an autophagy inhibitor by blocking autolysosome formation, synergistically enhanced the cytocidal effect in myeloma cell lines, compared with treatment with BZ or BAF alone (9). We therefore investigated whether the combination of BZ and CAM enhances cytotoxicity in breast cancer cells. Treatment of MDA-MB-231 cells with 5–100 μg/ml of CAM alone resulted in little growth inhibition (Fig. 3A). However, treatment with BZ in the presence of 25 and 50 μg/ml of CAM considerably enhanced cell growth inhibition in MDA-MB-231 and MDA-MB-468 cells (Fig. 3A and B). Morphological studies with May-Grünwald-Giemsa staining revealed that CAM treatment resulted in increased large vacuole formation in cytoplasm. Simultaneous treatment with CAM and BZ further resulted in high levels of vacuolization in cytoplasm, as well as nuclear chromatin condensation in the majority of cells (Fig. 4A). Electron microscopy also demonstrated that treatment with either BZ or CAM alone increased the number of autophagosomes, and combined treatment with BZ and CAM further increased the number of autophagosomes and autolysosomes (Fig. 4B).

**Figure 5:**
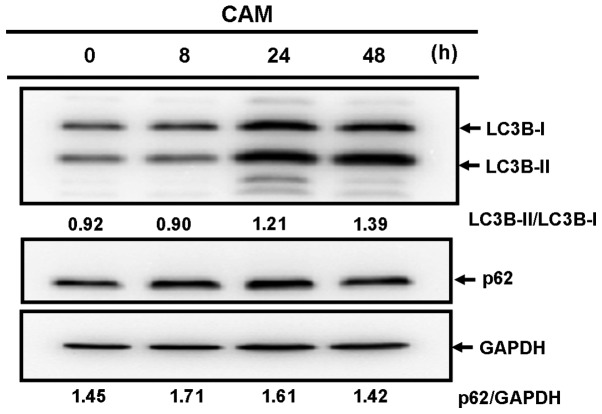

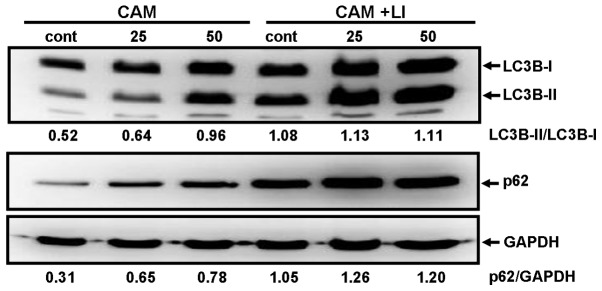
Immunoblotting with anti-LC3B Ab and anti-p62 Ab after treatment with CAM in MDA-MB-231 cells. (A) MDA-MB-231 cells were treated with CAM (50 μg/ml) for various lengths of time. (B) MDA-MB-231 cells were cultured with CAM (25 and 50 μg/ml) in the presence or absence of lysosomal inhibitors (LI), E-64d (10 μg/ml) and pepstatin A (10 μg/ml) for 48 h. Cellular proteins were separated by 15% SDS-PAGE for LC3B and 11.25% SDS-PAGE for p62, and immunoblotted with anti-LC3B Ab and anti-p62 mAb. Immunoblotting with anti-GAPDH mAb was performed as an internal control. Each number indicates the ratio of LC3B-II/LC3B-I and p62/GAPDH as determined by densitometry.

First, we examined whether the increased number of auto-phagosomes in response to CAM was due to the blocking of the catabolic process of autophagosomes or the inducement of autophagy (26). CAM treatment increased the ratio of LC3B-II to LC3B-I (Fig. 5A). Treatment with lysosomal inhibitors also increased this ratio by blocking the catabolic flux of autophagy. However, combined treatment with CAM and lysosomal inhibitors did not further increase the LC3B-I/LC3B-II ratio, compared with treatment with either CAM or lysosomal inhibitors alone (Fig. 5B). This result indicated that CAM blocks autophagy flux, but does not induce autophagy (24).

**Figure 6:**
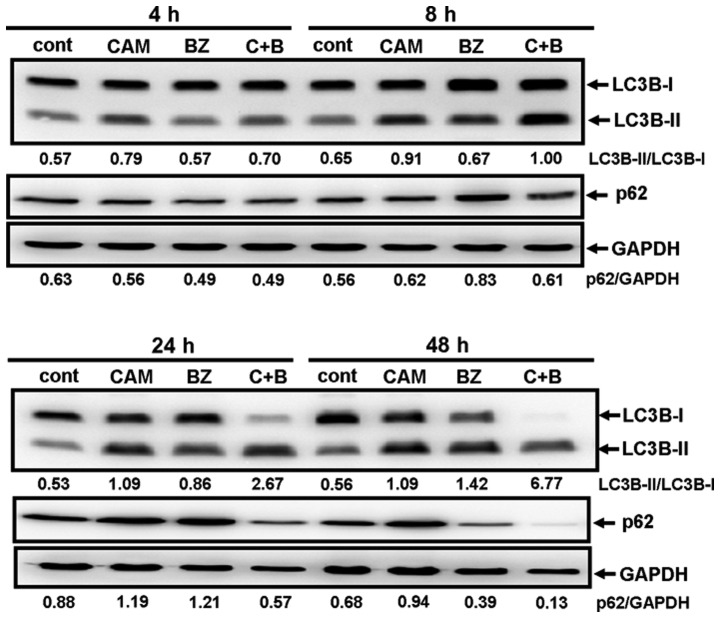

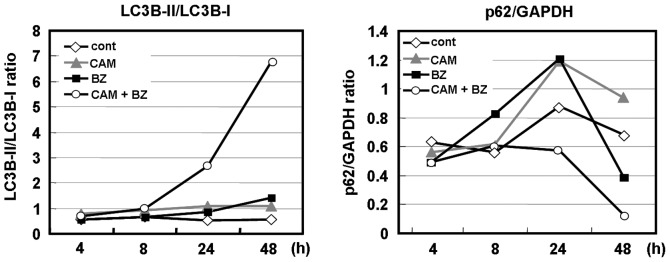
Immunoblotting with anti-LC3B Ab and anti-p62 Ab after combined treatment of MDA-MB-231 cells with CAM and BZ. (A) MDA-MB-231 cells were treated with/without CAM (50 μg/ml) in the presence or absence of BZ (25 nM) for various lengths of time. Cellular proteins were separated by 15% SDS-PAGE for LC3B and 11.25% SDS-PAGE for p62, then immunoblotted with anti-LC3B Ab and anti-p62 mAb. Immunoblotting with anti-GAPDH mAb was performed as an internal control. Each number indicates the ratio of LC3B-II/LC3B-I and p62/GAPDH as determined by densitometry. C + B indicates CAM + BZ. (B) Kinetics of the expression ratios of LC3B-I/LC3B-II and p62/GAPDH during 48-h treatment with/without BZ (25 nM) in the presence or absence of CAM (50 μg/ml).

Next, MDA-MB-231 cells were cultured with/without CAM in the presence/absence of BZ for various lengths of times and the ratios of LC3B-I/LC3B-II, as well as the expression levels of p62, were monitored. Although either CAM or BZ alone increased the LC3B-I/LC3B-II ratios, combined treatment with BZ and CAM for 24–48 h further increased the ratios (Fig. 6). This result agrees well with the light microscopic and the electron microscopic findings (Fig. 4). The pronounced clearance of p62 was also detected by combined treatment with CAM and BZ for 48 h (Fig. 6). These data indicate that CAM plus BZ enhances autophagy, compared with BZ alone.

### Involvement of CHOP induction for enhanced cytotoxicity by combined treatment with CAM and BZ

**Figure 7:**
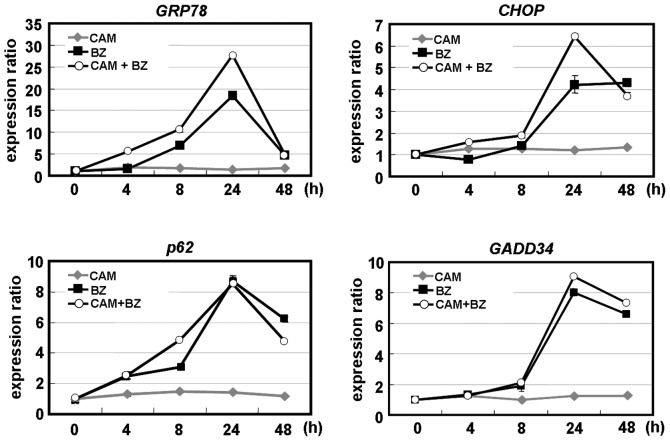

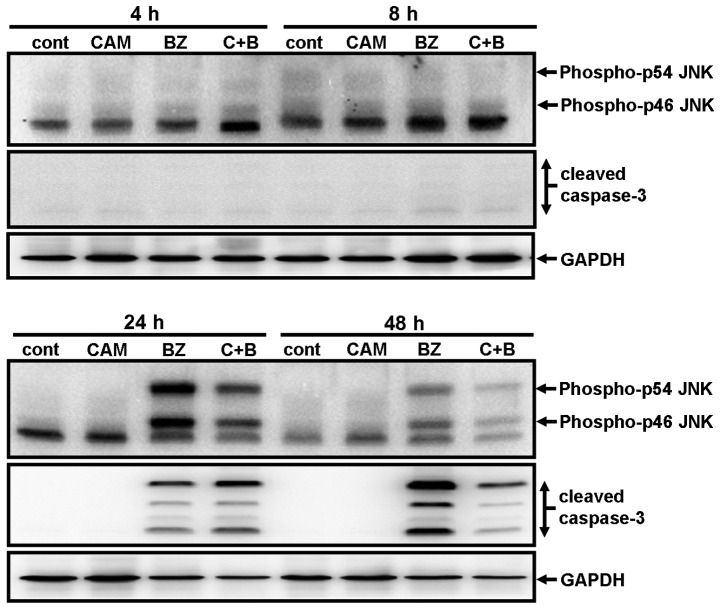
Induction of ER-stress-related genes and apoptosis in MDA-MB-231 cells after treatment with CAM and BZ. (A) Kinetics of GRP78, GADD34, CHOP and p62 expressions assessed by quantitative real-time PCR during 48-h exposure to CAM (50 μg/ml), BZ (25 nM), and CAM (50 μg/ml) + BZ (25 nM) in MDA-MB-231 cells. The data of the real-time PCR products for each gene were standardized to GAPDH as an internal control. The expression levels of GRP78, GADD34, CHOP and p62 were compared with those in untreated cells. (B) MDA-MB-231 cells were treated with/without CAM (50 μg/ml) in the presence or absence of BZ (25 nM) for various lengths of time. Cellular proteins were separated by 11.25% SDS-PAGE for phospho-JNK and 15% SDS-PAGE for cleaved caspase-3, and immunoblotted with anti-phospho-JNK (Thr183/Tyr185) Ab and anti-cleaved caspase-3 Ab. Immunoblotting with anti-GAPDH mAb was performed as an internal control. C + B indicates CAM + BZ.

To clarify the underlying molecular mechanism for cytotoxic enhancement by CAM plus BZ, we further investigated ER-stress induction. Real-time PCR indicated that the chaperone protein GRP78 and the pro-apoptotic transcription factor CHOP. CAM alone did not induce these ER-stress-mediated genes. However, the combination of CAM and BZ enhanced GRP78 and CHOP after 24-h treatment. An increased expression of GADD34, one of the genes transcriptionally regulated by CHOP, was observed with the combination of CAM and BZ (13). Notably, BZ, but not CAM, induced p62 mRNA. BZ plus CAM did not further increase p62 gene expression, compared with BZ alone (Fig. 7A). This result appeared to reflect the transient increase of p62 protein expression after 48-h treatment with BZ (Fig. 2A and B), because intracellular protein expression is determined by dynamic equilibrium between synthesis and degradation. Immunoblottings detected phosphorylation of JNK, as well as cleaved caspase-3, after treatment with BZ alone and BZ plus CAM (Fig. 7B).

**Figure 8:**
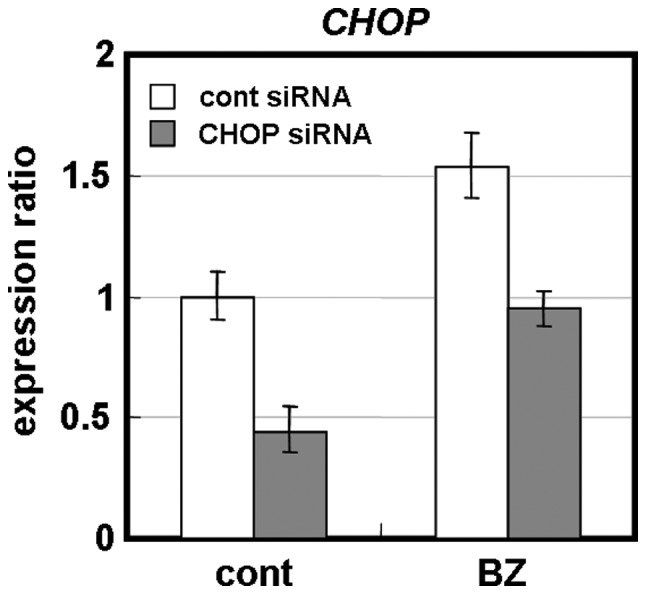

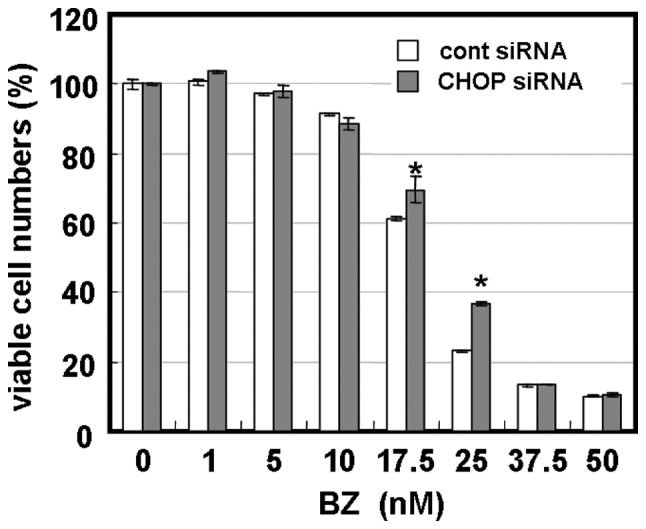
Cell growth inhibition by BZ after knockdown of CHOP by siRNA in MDA-MB-231 cells. (A) MDA-MB-231 cells were pretreated with either siRNA for CHOP or control siRNA for 48 h as described in Materials and methods. Expression of CHOP mRNA was assessed by real-time PCR. (B) After 48-h treatment with siRNA, cells were subsequently treated with BZ at various concentrations for 48 h and the viable number of cells was assessed by Cell TiterBlue as described in Materials and methods (^*^p<0.05, control siRNA vs. CHOP siRNA).

**Figure 9:**
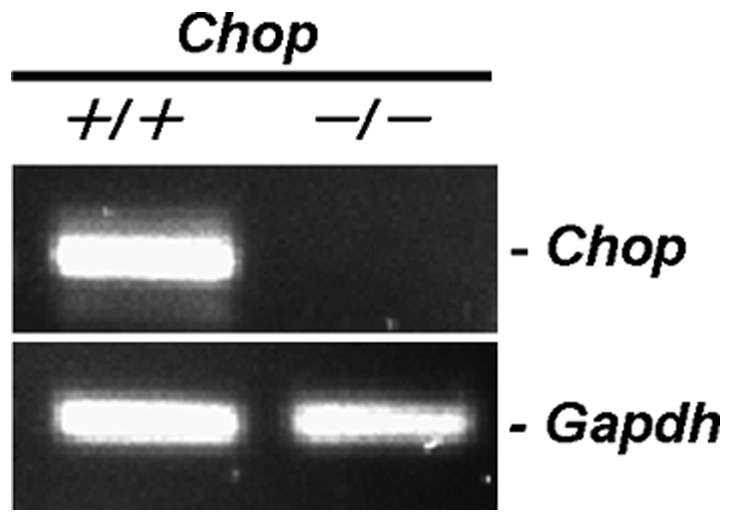

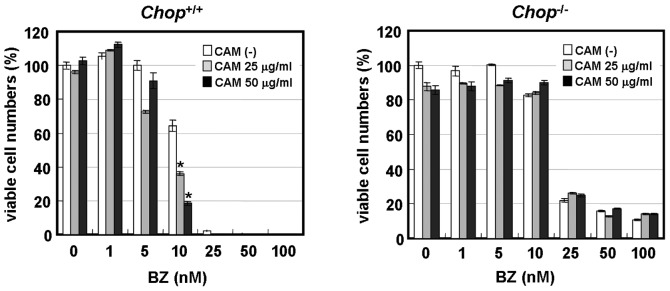
Cell growth inhibition of CHOP^−/−^ MEF cell line and wild-type MEF cell line after treatment with BZ in the presence or absence of CAM. (A) Gene expression analysis of CHOP in wild-type CHOP^+/+^ MEF and CHOP^−/−^ MEF cell line by RT-PCR. The sequences of primes for *Chop* and *Gapdh* were as we previously described (38). (B) CHOP^−/−^ MEF cell line and CHOP^+/+^ MEF cell line were cultured in the presence of BZ at various concentrations with/without CAM (25 and 50 μg/ml) for 48 h. The number of viable cells was assessed as described in Materials and methods [^*^p<0.05, CAM (−) vs. CAM (+)].

It has been suggested that ER-stress-mediated CHOP induction exerts cytotoxicity of BZ in myeloma cells (7–10). Transient knockdown for CHOP by siRNA in MDA-MB-231 cells appeared less sensitive to BZ than control siRNA-treated cells (Fig. 8). Furthermore, CHOP^−/−^ MEF cells were more resistant to BZ than wild-type MEF cells (Fig. 9). Combined treatment with CAM and BZ resulted in pronounced cytotoxicity in wild-type MEF cells, whereas this enhancement was completely cancelled in CHOP^−/−^ MEF cells. All these data suggest that ER-stress-mediated CHOP induction was involved in the cytotoxic effect of BZ in breast cancer cell lines. Additionally, enhanced cytotoxicity by combining two reagents appeared to be due to increased CHOP induction via UPR.

## Discussion

In the present study, we demonstrated that combined treatment with BZ and CAM enhanced cytotoxicity in breast cancer cells (Fig. 3A and B). Treatment with CAM alone had little effect on cell growth inhibition and UPR; however, simultaneous treatment with BZ plus CAM enhanced UPR, leading to induction of the transcription factor CHOP and the chaperone protein GRP78 in MDA-MB-231 cells (Fig. 7). Notably, this enhancement was completely cancelled in CHOP^−/−^ MEF cells, whereas wild-type MEF cells clearly exhibited pronounced cytotoxicity with BZ plus CAM, as observed in MDA-MB-231 cells (Fig. 9). Therefore, it is strongly suggested that CHOP is involved in enhanced cytotoxicity. It has been reported that CHOP not only transcriptionally induces pro-apoptotic proteins such as Bim, BAX, and DR5 but also down-regulates the anti-apoptotic protein Bcl-2 (13,39). Therefore, the profile of all downstream gene expressions in response to CHOP appears to direct the cells to undergo apoptosis (13,39).

Treatment of MDA-MB-231 cells with CAM increased LC3B-II/LC3B-I ratios. In the presence of lysosomal inhibitors, these ratios did not increase any further with the accumulation of p62 (Fig. 4). These data suggest that the increased number of autophagosomes in cytoplasm in response to CAM, as observed in light and electron microscopy, is caused by the accumulation of autophagosomes by blocking autophagy flux, but not by the induction of autophagy. It has been reported that treatment with CAM attenuates autophagy by blocking the late phase of the autophagic process, probably after the fusion of autophagosomes with lysosomes in myeloma cells (26). It has also been reported that, in addition to proteasome-mediated degradation, the ubiquitinated proteins are selectively degraded by autophagy via the docking protein p62, which has both an LC3-interacting region and a ubiquitin-binding domain (23). Therefore, it makes sense that blocking two major proteolytic pathways, such as the ubiquitin-proteasome system by BZ and the autophagy-lysosome system by CAM, results in the accumulation of unfolded proteins in ER and subsequently the induction of CHOP. We have reported on a similar phenomenon in myeloma cells using BZ and the autophagy inhibitor bafilomycin A_1_ (9). However, previous reports demonstrated that inhibiting formation of autophagosome by either 3-methyladenine or siRNA for LC3 or knockout of *atg5*gene, somewhat attenuated BZ-induced cytotoxicity in myeloma cells (9,40). We cannot clearly explain this discrepancy. All these treatments inhibit autophagosome formation, whereas treatment with either CAM or bafilomycin A_1_ inhibits the late stage of autophagy, such as inhibiting autolysosome formation or lysosomal hydrolysis (9,26). Further studies will be required to assess the intracellular loading volume for ER-stress by blocking autophagosome formation, and by blocking autolysosome formation or lysosomal hydrolysis. Recent reports demonstrated that the non-canonical autophagic pathway does not require the entire set of autophagy-related (Atg) proteins (41,42). Therefore, an alternative pathway to form autolysosome may provide a bypass to avoid loading ER-stress, even though the canonical autophagy pathway is blocked.

Some contradiction still exists regarding autophagy induction in response to BZ. It has been reported that BZ blocks the catabolic process of autophagy via a cathepsin-dependent mechanism in estrogen receptor-positive breast cancer cells (16), whereas other reports demonstrated that BZ induces autophagy in myeloma cells, prostatic cancer cells, endothelial cells, and breast cancer cells (9,15,40,43,44). BZ has been reported to induce autophagy via proteasomal stabilization of activating transcription factor 4 (ATF4) and up-regulation of LC3B by ATF4, thus preventing BZ-induced cell death in MCF7 breast cancer cells (15). ATF4 is a transcription factor reported to be induced under severe hypoxia and a component of the PERK pathway involved in the UPR (45). ATF4 facilitates autophagy through direct binding to a cyclic AMP response element-binding site in the LC3B promoter, resulting in LC3B up-regulation (45,46). It has also been reported that ER-stress evokes up-regulation of the transcriptional co-activator p8 and its target, the pseudo-kinase tribbles homolog 3 (TRB3), and subsequently induced autophagy via inhibition of the Akt/mTORC1 axis in human glioma cells (47). These data indicate a direct signaling pathway from ER-stress to autophagy induction. Therefore, autophagy induction in response to BZ may occur via ER-stress-mediated AFT4 induction. In addition, enhanced autophagy induction by combined treatment with CAM and BZ might be explained by ATF4 mediate autophagy during ER-stress loading. A similar phenomenon was previously reported regarding enhanced cytotoxicity by the combination of BZ and an inhibitor for the autophagy-lysosome pathway in myeloma cells (48). The microtubule-organizing center (MTOC) transports unfolded proteins to lysosomes and are degraded through the autophagy-lysosome pathway. Histone deacetylase 6 (HDAC6) deacetylates α-tubulin, which is thought to be a component of the MTOC. HDAC6 knockdown results in decreased LC3B protein and reduces autophagy (49). Tubacin, a small molecule inhibitor of HDAC6, prevents deacetylation of α-tubulin and produces accumulation of polyubiquitinated proteins and apoptosis, and further acts synergistically with BZ to induce cytotoxicity in multiple myeloma (48). Based on our results presented here, these data also can be explained by enhanced loading on ER-stress by simultaneously targeting the autophagy-lysosome pathway by tubacin and the ubiquitin-protease pathway by BZ.

One may ask whether the enhanced cytotoxicity archived by combining BZ and CAM is simply due to the pharmacointeraction between CYP3A4 and CAM. CAM is known to inhibit CYP3A4, the isoenzyme responsible for metabolizing BZ (50,51). Therefore, concomitant administration of BZ with CAM may lead to intracellular elevation of BZ concentrations. We cannot completely exclude this possibility. However, it is unlikely because combined treatment with CAM and either docetaxel or paclitaxel, both of which are chemotherapeutic reagents for breast cancer and are metabolized by CYP3A4 as well as BZ, did not result in any enhanced cytotoxicity in MDA-MB-231 cells, compared with treatment with each reagent alone (data not shown).

As indicated in Figs. 2 and 7A, p62 was transiently increased in response to BZ after 24–48-h treatment. P62 is a stress response protein that is strongly induced at the mRNA and protein levels by exposure to oxidants, sodium arsenite, cadmium, ionophores, and proteasome inhibitors (52). Interestingly, p62 is over-abundant in malignant breast tissue, compared with normal breast tissue (53). The proteasome inhibitor PSI has been reported to increase p62 mRNA and protein as well as BZ does (52). Since the intracellular protein level is determined by dynamic equilibrium between protein synthesis and degradation, a transient increase in response to BZ appears to reflect the predominance of p62 synthesis at that time-point. However, abundance of intracellular p62 in breast cancer cells, which may be caused by some intrinsic stress response, may determine sensitivity to BZ (53). We are now in the process of clarifying the molecular mechanism in order to explain why MCF7 cells are less sensitive to BZ than MDA-MB-231 and MDA-MB-468 cells (Fig. 1). This will identify the determinant factor for indicating BZ-therapy in breast cancer patients.

Considering all the data, ER-stress-mediated CHOP appears to be involved in the cytotoxicity of BZ in breast cancer cells. Combining BZ and CAM is suggested to be a promising option for improving the therapeutic outcome of breast cancer therapy. Additionally, simultaneously targeting the ubiquitin-proteasome pathway and the autophagy-lysosome pathway appears to be a rational strategy to enhance chemo-sensitivity and overcome chemo-resistance in cancer therapy.
